# Association between potassium concentrations, variability and supplementation, and in-hospital mortality in ICU patients: a retrospective analysis

**DOI:** 10.1186/s13613-019-0573-0

**Published:** 2019-09-05

**Authors:** Lilian Jo Engelhardt, Felix Balzer, Michael C. Müller, Julius J. Grunow, Claudia D. Spies, Kenneth B. Christopher, Steffen Weber-Carstens, Tobias Wollersheim

**Affiliations:** 1 Department of Anesthesiology and Operative Intensive Care Medicine (CCM, CVK), Charité–Universitätsmedizin Berlin, corporate member of Freie Universität Berlin, Humboldt-Universität zu Berlin, and Berlin Institute of Health, Augustenburger Platz 1, 13353 Berlin, Germany; 2 Division of Renal Medicine, Brigham and Women’s Hospital, Harvard Medical School, 75 Francis Street, Boston, MA USA; 3 Berlin Institute of Health (BIH), Anna-Louisa-Karsch-Str. 2, 10178 Berlin, Germany

**Keywords:** Potassium, Potassium supplementation, Potassium variability, Potassium target, ICU, Critically ill patients, Mortality

## Abstract

**Background:**

Serum potassium concentrations are commonly between 3.5 and 5.0 mmol/l. Standardised protocols for potassium range and supplementation in the ICU are lacking. The purpose of this retrospective analysis of ICU patients was to investigate potassium concentrations, variability and supplementation, and their association with in-hospital mortality.

**Methods:**

ICU patients ≥ 18 years, with ≥ 2 serum potassium values, treated at the Charité - Universitätsmedizin Berlin between 2006 and 2018 were eligible for inclusion. We categorised into groups of mean potassium concentrations: < 3.0, 3.0–3.5, > 3.5–4.0, > 4.0–4.5, > 4.5–5.0, > 5.0–5.5, > 5.5 mmol/l and potassium variability: 1st, 2nd and ≥ 3rd standard deviation (SD). We analysed the association between the particular groups and in-hospital mortality and performed binary logistic regression analysis. Survival curves were performed according to Kaplan–Meier and tested by Log-Rank. In a subanalysis, the association between potassium supplementation and in-hospital mortality was investigated.

**Results:**

In 53,248 ICU patients with 1,337,742 potassium values, the lowest mortality (3.7%) was observed in patients with mean potassium concentrations between > 3.5 and 4.0 mmol/l and a low potassium variability within the 1st SD. Binary logistic regression confirmed these results. In a subanalysis of 22,406 ICU patients (ICU admission: 2013–2018), 12,892 (57.5%) received oral and/or intravenous potassium supplementation. Potassium supplementation was associated with an increase in in-hospital mortality in potassium categories from > 3.5 to 4.5 mmol/l and in the 1st, 2nd and ≥ 3rd SD (*p* < 0.001 each).

**Conclusions:**

ICU patients may benefit from a target range between 3.5 and 4.0 mmol/l and a minimal potassium variability. Clear potassium target ranges have to be determined. Criteria for widely applied potassium supplementation should be critically discussed.

*Trial registration* German Clinical Trials Register, DRKS00016411. Retrospectively registered 11 January 2019, http://www.drks.de/DRKS00016411

## Background

Potassium homeostasis is regulated within extracellular concentrations between 3.5 and 5.0 mmol/l [1]. This is of importance for physiological processes, such as the negative resting membrane potential and consequently neuromuscular and cardiac excitability [1]. Severe hyperkalaemia is associated with ventricular arrhythmia, bradycardia and cardiac arrest [2]. Symptoms of severe hypokalaemia include muscle weakness up to paralysis, respiratory insufficiency, constipation and cardiac arrhythmias [3]. Hypo- and hyperkalaemia are associated with an increased complication rate and mortality risk [1–6].

Contributing mechanisms for potassium electrolyte disorders include disturbances in potassium ingestion, excretion and potassium shifting between intra- and extracellular compartment. Various medications interfere with potassium homeostasis [3]. The drug with the highest risk for inducing hyperkalaemia is the intravenous potassium supplementation [7].

The European Resuscitation Council Guidelines for Resuscitation 2015 state that there is no universal definition of hyperkalaemia [2]. They define hypokalaemia as serum potassium concentration < 3.5 mmol/l and hyperkalaemia as > 5.5 mmol/l. According to the guideline, therapy to lower potassium concentration should be initiated > 5.5 mmol/l. Severe hypokalaemia should be treated with intravenous potassium application in emergency situations [2]. In patients with myocardial infarction, a previous American College of Cardiology/American Heart Association (ACC/AHA) Guideline suggested to elevate potassium concentrations > 4.0 mmol/l to reduce the risk of ventricular fibrillation [8, 9]. Evidence for potassium targets in the upper reference level originates from studies implemented before the routine use of β-blockers and reperfusion therapy in patients with myocardial infarction [8]. Potassium repletion to 4.0 mmol/l or higher is recommended in patients with torsade de pointes episodes associated with medication-induced QT prolongation [10]. After cardiac and thoracic surgery, guidelines recommend to correct hypokalaemia to 4.5–5.5 mmol/l in order to prevent atrial fibrillation, although they emphasise that this suggestion has never been scientifically confirmed [11]. In summary, previous guidelines recommend potassium concentrations rather in the upper normokalaemic range. However, there is evidence that potassium concentrations above 4.5 mmol/l may be adversely associated with survival in patients with myocardial infarction [8, 12].

Increased mortality has been shown for hyperkalaemia in ICU patients [6]. To our knowledge, there is no valid guideline available regarding potassium target ranges and supplementation in ICU patients. The purpose of this retrospective study was to analyse mean potassium concentrations as well as potassium variability and their association with mortality in a general ICU population. We aimed to identify possible mortality differences within the normal range. Until now, this is the largest analysis considering the association between mortality and potassium concentrations in the range from 3.0 to > 5.5 mmol/l. In addition, we investigated the association of potassium supplementation with mortality in groups of potassium mean and variability.

## Materials and methods

We performed a retrospective analysis of ICU patients treated in the Department of Anaesthesiology and Operative Intensive Care Medicine, Charité – Universitätsmedizin Berlin. Trial registration: German Clinical Trials Register, DRKS00016411. Retrospectively registered 11 January 2019, http://www.drks.de/DRKS00016411.

### Inclusion criteria and patient cohort

ICU patients $\ge$ 18 years with a minimum of two serum potassium values during ICU stay who were treated in one of the ICUs of the Department of Anaesthesiology and Operative Intensive Care Medicine, Charité – Universitätsmedizin Berlin between January 2006 and May 2018 were eligible for inclusion. Potassium values > 10 mmol/l and < 2 mmol/l were excluded.

### Data sources and ethics vote

We extracted data from the local patient documentation system. All patient data including blood gas analysis, laboratory measurements, medications, patient baseline characteristics and vital signs are documented in this patient data management system. The retrospective analysis of these data was approved by the local ethics committee, Ethikkommission Charité - Universitätsmedizin Berlin, Germany (EA2/187/18).

### Exposure of interest and covariates

The exposure of interest was the patient’s mean potassium value during the ICU stay. Patients were categorised into groups of mean potassium: < 3.0, 3.0–3.5, > 3.5–4.0, > 4.0–4.5, > 4.5–5.0, > 5.0–5.5 and > 5.5 mmol/l. In addition, similar analyses were performed in categories of first potassium values at ICU admission. Potassium variability was measured in (a) standard deviation (SD) of serum potassium measurements per patient, classified into 1st, 2nd and ≥ 3rd SD groups and (b) coefficient of variation (CV) (SD/mean potassium), classified into 4 groups: CV1 0–10%, CV2 10–20%, CV3 20–30%, CV4 > 30%. Finally, we combined groups of mean potassium and variability and analysed in-hospital mortality risk. Potassium values were determined by blood gas analyser (Radiometer ABL 600 or 800 FLEX, Copenhagen, Denmark) or laboratory standard. Investigated covariates were age, gender, mean blood glucose concentration, blood glucose variability measured as SD, minimum and maximum blood glucose concentration, mean sodium concentration, sodium SD, acute physiology and chronic health evaluation (APACHE) II score, mean maximum sepsis-related organ failure assessment (SOFA) score, diabetes, acute kidney injury, chronic kidney disease, atrial fibrillation, length of stay in the ICU, number of potassium measurements and pH value. APACHE II score was evaluated at ICU admission whilst SOFA score was determined on a daily basis by physicians. Covariates identified by ICD-codes like hypokalaemia, hyperkalaemia, chronic kidney disease, acute kidney injury, diabetes, arterial hypertension and atrial fibrillation were taken from the patients’ hospital records. ICD-10-GM derived covariates are listed in Additional file 1.

### Endpoint

The primary endpoint was all-cause in-hospital mortality. Information on vital status for the study cohort was obtained from hospital records. The censoring date was May 31, 2018.

### Statistical analysis

We used IBM© SPSS© Statistics version 24, Microsoft^®^ Excel and SigmaPlot version 12.5 and GraphPad Prism 7 for data analysis and visualisation. Results are expressed as median with interquartile range or as absolute numbers with percentages. For the variables potassium, glucose, sodium and pH values, we calculated mean values for each patient and reported median and 25th/75th percentile of the cohort. A *p* value < 0.05 was considered statistically significant. For the statistical analysis of group differences, we performed Mann–Whitney *U* test, Kruskal–Wallis test and *χ*^2^ test. To verify confounding factors, we performed a binary logistic regression analyses stepwise unadjusted, adjusted for sex, age and mean pH value, and adjusted for clinically important possible confounders as listed above. 20-fold imputations and rerun of the binary logistic regression analyses was performed to confirm the results in spite of missing values. Survival curves for mean potassium range and potassium SD groups were performed according to Kaplan–Meier and tested by Log-Rank-test.

### Potassium supplementation and in-hospital mortality

In this subanalysis, we included only patients from our cohort treated between 2013 and 2018 with medication records available to compare in-hospital mortality risk of patients with and without potassium supplementation. Potassium supplementation included oral and/or intravenous substitution for at least one time. In-hospital mortality analyses were performed in each group of mean potassium concentration and potassium variability.

## Results

We included 53,248 ICU patients with overall 1,337,742 serum potassium values. 7.2% of these patients died during hospital stay (*n* = 3823). Detailed information on patient’s baseline characteristics is shown for groups of mean potassium (Table 1) and for groups of variability (Additional file 1: Table S1). After exclusion of potassium measurements, < 2 mmol/l no patient presented with mean potassium concentrations of < 3.0 mmol/l.

**Table 1:** Baseline characteristics in categories of mean potassium range, *n* = 53,248 patients

Characteristics	3.0–3.5 mmol/l	> 3.5–4.0 mmol/l	> 4.0–4.5 mmol/l	> 4.5–5.0 mmol/l	> 5.0–5.5 mmol/l	>5.5 mmol/l
Number of patients	3333	20,790	20,599	7312	950	264
Age	64 [51/74]	63 [50/74]	67 [56/75]	69 [60/76]	65 [54/75]	60 [48/71]
Gender (%)						
Female/male	2249 (67.5)/1084 (32.5)	10,904 (52.4)/9886 (47.6)	7739 (37.6)/12,860 (62.4)	2303 (31.5)/5009 (68.5)	313 (32.9)/637 (67.1)	85 (32.2)/179 (67.8)
APACHE II score	11.00 [5.00/17.00]	11.00 [5.00/16.00]	12.00 [6.00/19.00]	15.00 [8.00/22.00]	14.00 [5.00/21.00]	15.00 [4.00/23.00]
SOFA score (mean)	1.40 [0.40/3.00]	1.50 [0.33/2.89]	2.00 [0.50/3.75]	2.90 [1.33/4.63]	2.50 [0.50/4.51]	2.50 [0.00/5.29]
SAPS II	26.00 [14.00/37.00]	25.00 [13.00/36.00]	29.00 [17.00/41.00]	34.00 [22.00/50.00]	31.00 [14.00/44.00]	29.00 [13.00/48.00]
Length of ICU stay (h)	23.95 [15.25/69.45]	25.90 [16.60/75.90]	45.90 [19.30/138.60]	85.20 [23.50/167.90]	27.90 [14.80/85.25]	22.45 [10.40/74.15]
Mean glucose level (mg/dl) *n* = 43,694	116.00 [100.50/138.50]	117.67 [102.50/138.02]	126.00 [108.07/148.70]	135.86 [117.41/158.00]	125.36 [105.00/156.00]	116.50 [92.77/150.50]
Mean glucose SD (mg/dl) *n* = 33,031	17.79 [9.19/29.70]	19.09 [11.02/30.37]	23.50 [14.00/35.70]	29.02[20.23/42.33]	26.06 [16.28/40.88]	19.05 [7.82/39.92]
Glucose min (mg/dl) *n* = 43,694	103.00 [88.00/123.00]	99.00 [87.00/118.00]	97.00 [84.00/118.00]	91.00 [77.00/109.00]	91.50 [77.00/122.00]	93.00 [74.50/127.00]
Glucose max (mg/dl) *n* = 43,694	129.00 [107.00/159.00]	137.00 [112.00/172.00]	157.00 [121.00/210.00]	193.00 [144.00/252.00]	164.00 [125.00/216.00]	138.00 [106.50/187.50]
Sodium mean (mmol/l)	139.20 [137.00/141.00]	139.00 [137.20/140.82]	138.75 [136.92/140.50]	138.50 [136.68/140.16]	137.77 [135.98/139.63]	137.48 [135.71/139.10]
ph value mean *n* = 30,558	7.41 [7.38/7.45]	7.40 [7.37/7.43]	7.40 [7.37/7.43]	7.39 [7.36/7.41]	7.35 [7.31/7.39]	7.33 [7.24/7.37]
Patients with mechanical ventilation (%)	603/3319 (18.2)	4026/20,721 (19.4)	7111/20,515 (34.7)	3450/7275 (47.4)	284/948 (30.0)	80/263 (30.4)
Patients receiving renal replacement therapy (%)	93/3319 (2.8)	798/20,721 (3.9)	1995/20,515 (9.7)	1457/7275 (20.0)	394/948 (41.6)	126/263 (47.9)
ICD-10 diagnoses number of patients with positive diagnosis/absolute numbers (in %)						
Diabetes mellitus	1133/3333 (34.0)	6591/20,790 (31.7)	8234/20,599 (40.0)	3597/7315 (49.2)	424/950 (44.6)	98/264 (37.1)
AKI	275/3333 (8.3)	1632/20,784 (7.9)	3333/20,592 (16.2)	1774/7309 (24.3)	242/950 (25.5)	76/264 (28.8)
CKD	336/3333 (10.1)	2848/20,784 (13.7)	5151/20,592 (25.0)	2951/7309 (40.4)	674/950 (70.9)	151/264 (57.2)
Atrial fibrillation	497/3333 (14.9)	3557/20,784 (17.1)	5521/20,592 (26.8)	2751/7309 (37.6)	262/950 (27.6)	59/264 (22.3)
Hypertension	1692/3333 (50.8)	10,027/20,784 (48.2)	12,043/20,592 (58.5)	5031/7309 (68.8)	631/950 (66.4)	141/264 (53.4)
Hypokalaemia	2217/3333 (66.5)	9313/20,784 (44.8)	8075/20,592 (39.2)	2412/7309 (33.0)	148/950 (15.6)	37/264 (14.0)
Hyperkalaemia	39/33,333 (1.2)	279/20,784 (1.3)	934/20,592 (4.5)	930/7309 (12.7)	330/950 (34.7)	115/264 (43.6)

Groups were categorised by means of potassium concentrations. Results are expressed as median with interquartile range or as absolute numbers with percentages. Missing values/patients are < 2% and considered as not relevant or indicated. Glucose values are available in *n* = 43,694 patients; glucose variability was determined in *n* = 33,031 patients. pH values are available in *n* = 30,558 patients. All presented parameters show significant differences in distribution over the categories with *p* < 0.001 tested by Kruskal–Wallis test

*SOFA* sepsis-related organ failure assessment, *APACHE* acute physiology and chronic health evaluation II score at ICU admission, *SAPS* simplified acute physiology score at ICU admission, *ICU* intensive care unit, *AKI* acute kidney injury, *CKD* chronic kidney disease

### In-hospital mortality in groups of mean potassium, first potassium value and variability

**Fig. 1:**
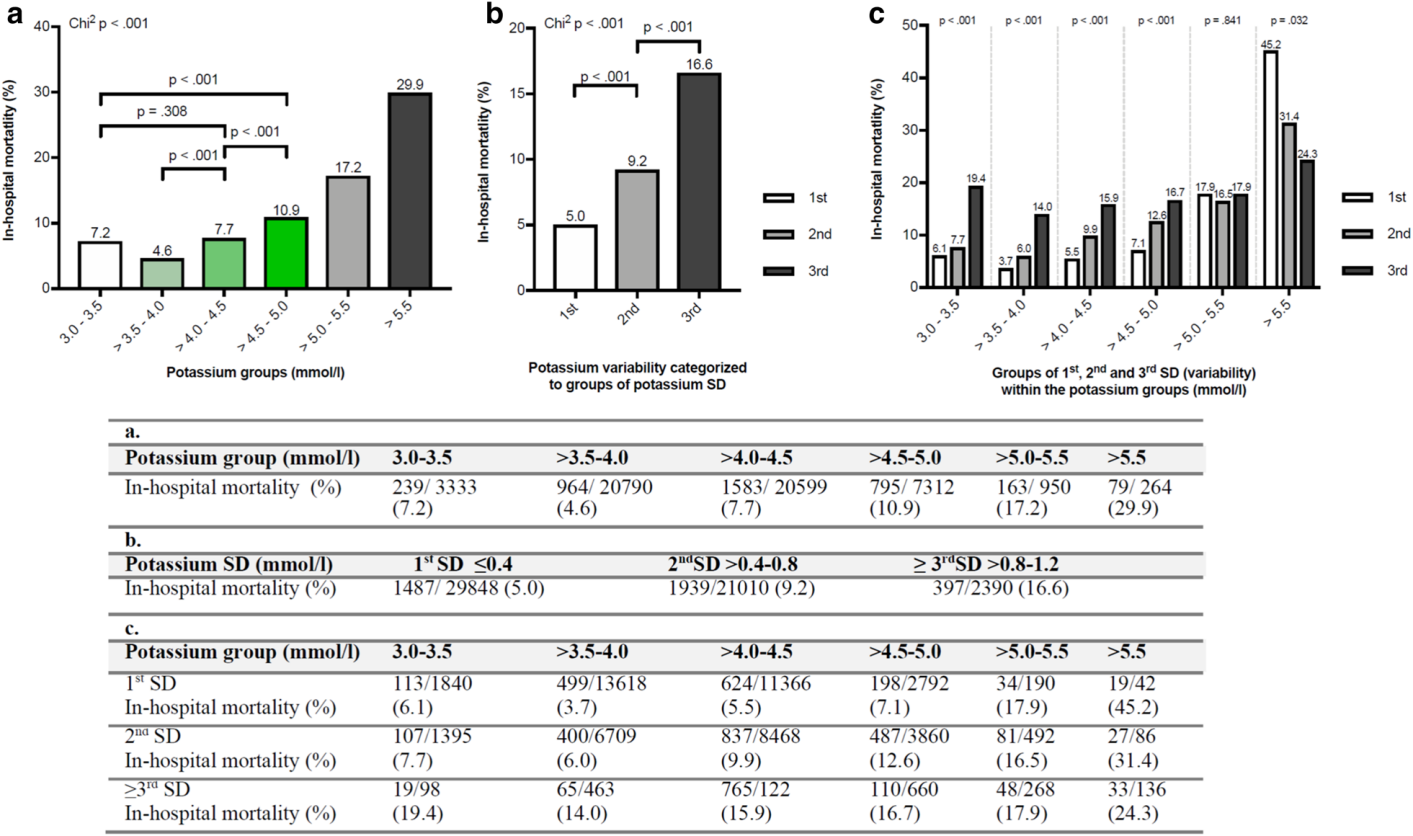
Association between mean potassium and variability groups and in-hospital mortality, *n* = 53,248 patients. The table shows the absolute number of patients and the number of deaths in each group. *χ*^2^ test for all groups, Fisher Exact test for comparison between subgroups. **a** In-hospital mortality rate is lowest in potassium range > 3.5–4.0 mmol/l. **b** Groups of potassium variability shown in 1st, 2nd and ≥ 3rd SD of serum potassium. In-hospital mortality is lowest in the 1st SD and increases in each group. *χ*^2^ test for all groups and comparison between subgroups. **c** Lowest in-hospital mortality rate (3.7%) was observed in patients with low variability (1st SD) and mean potassium concentrations > 3.5–4.0 mmol/l. Groups within the potassium normal range (3.5–5.0 mmol/l) are coloured green. *p* values determined by *χ*^2^-test

Median potassium concentrations per patient was 4.05 mmol/l [3.80/4.35], and median number of potassium measurements was 7 [3/18]. We found a J-curved association between mean potassium concentrations and in-hospital mortality with the lowest mortality risk in the potassium range from > 3.5 to 4.0 mmol/l (Fig. 1a). This result was also present using smaller cut points with a range of 0.1 mmol/l (Additional file 1: Figure S1). Mortality significantly increased within the normokalaemic range (*p* < 0.001, Fig. 1a). Mortality in the range of 3.0–3.5 mmol/l was significantly lower (*p* < 0.001, Fisher test) than between > 4.5 and 5.0 mmol/l (Fig. 1a). In a subanalysis, 12,647 patients with atrial fibrillation showed lowest mortality between > 3.5 and 4.0 mmol/l (Additional file 1: Figure S2). The distribution was different in patients receiving renal replacement therapy (Additional file 1: Figure S3). Considering the first potassium values at ICU admission, we identified a nadir of mortality (5.6%) in patients with initial potassium value of > 3.5–4.0 mmol/l (Additional file 1: Figure S4).

Mean of standard deviation (potassium variability) per patient was 0.4 mmol/l. An increase in potassium variability per patient to two or threefold was associated with a significant increase of in-hospital mortality rate (Fig. 1b). In an analysis, using smaller cut points with a range of 0.1 mmol/l a potassium variability between 0.1 and 0.3 mmol/l was associated with the lowest mortality (Additional file 1: Figure S5). In agreement, mortality increased significantly within the groups of CV (Additional file 1: Figure S6). The combination of mean potassium concentrations between 3.5 and 4.0 mmol/l and a low variability were associated with the lowest mortality (Fig. 1c: 1st SD mortality 3.7%; Additional file 1: Figure S7: CV group 1 mortality 3.6%).

Mean range and variability showed both an association with in-hospital mortality in a consistent regression.

### Probability of survival

**Fig. 2:**
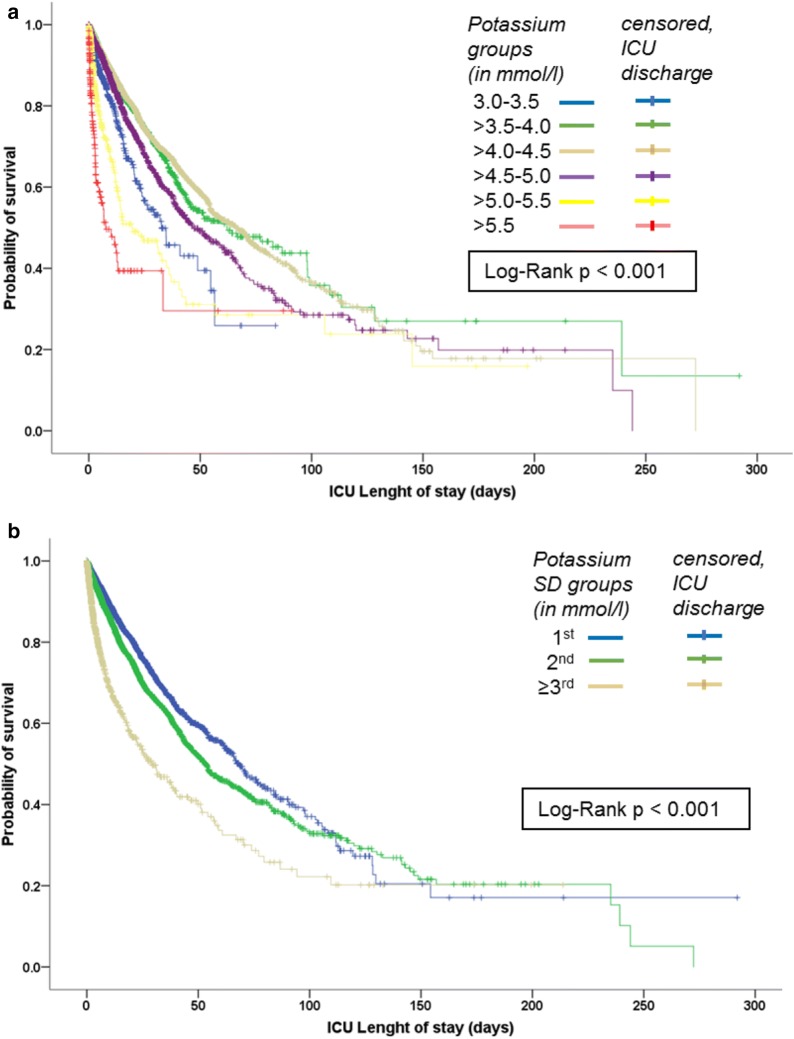
Kaplan–Meier curves. Survival curves show probability of survival at hospital discharge for (**a**) mean potassium groups and (**b**) potassium variability groups (in SD), tested by Log-Rank test

Probability of survival was significantly different in mean potassium groups and groups of potassium variability. Mean potassium values between 3.5 and 4.5 mmol/l as well as a low potassium variability within the 1st SD were associated with higher probability of survival in the Kaplan–Meier Curves (Fig. 2).

### Regression analysis

**Fig. 3:**
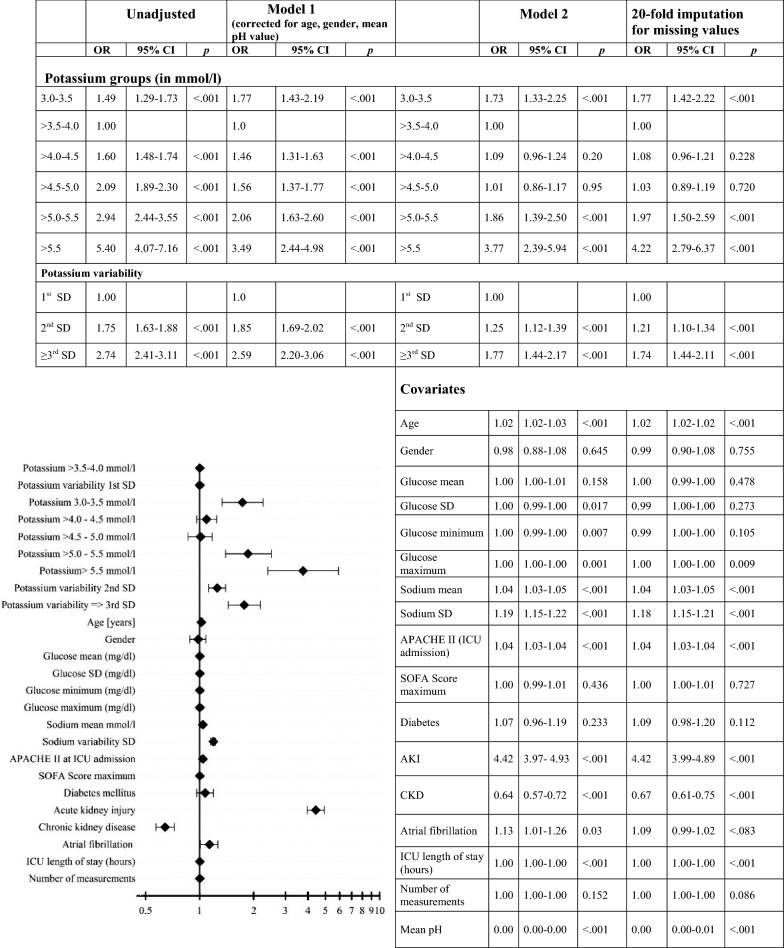
Adjusted odds ratios for in-hospital mortality. Reference for potassium categories is > 3.5–4.0 mmol/l, for potassium variability 1st SD. Unadjusted: *n* = 53,248 patients. Adjusted model 1: *n* = 30,558 patients, 22,690 missing values adjusted for gender, age and pH value. Adjusted model 2: *n* = 25,636 patients, 27,612 missing values, adjusted for gender, age, glucose mean, glucose SD, glucose maximum, glucose minimum, sodium mean, sodium SD, APACHE II score, SOFA score maximum, diabetes, acute kidney injury (AKI), chronic kidney disease (CKD), atrial fibrillation, number of measurements, ICU length of stay (in hours) and mean pH value; corrected for missing values by 20-fold imputations. Blood glucose concentrations in mg/dl and sodium concentration in mmol/l. Forest plot of original data from model 2

Increasing values for mean potassium and variability were independently associated with increased in-hospital mortality risk in the binary logistical regression analysis unadjusted and adjusted within model 1 for age, gender and pH value (Fig. 3 and Additional file 1: Table S2). In the model 2 adjusting stepwise for several clinical relevant confounders, the results differed after adding pH value to this model (Fig. 3 and Additional file 1: Figure S8 without mean pH). Categorising model 2 into groups of pH value (< 7.36, 7.36–7.44, > 7.44), mean potassium levels 3.5–4.0 mmol/l were independently associated with low mortality after adjusting for multiple confounders in the group of pH value < 7.36 (Additional file 1: Table S3). 1st SD was associated with decreased mortality after adjustment in each model. Within the model 2 with several confounders, the 20-fold imputation showed the same results as the original data set and consequently excluded an impact of missing values. Regression model 2 was visualised using a Forest plot (Fig. 3).

### Potassium supplementation and in-hospital mortality

**Fig. 4:**
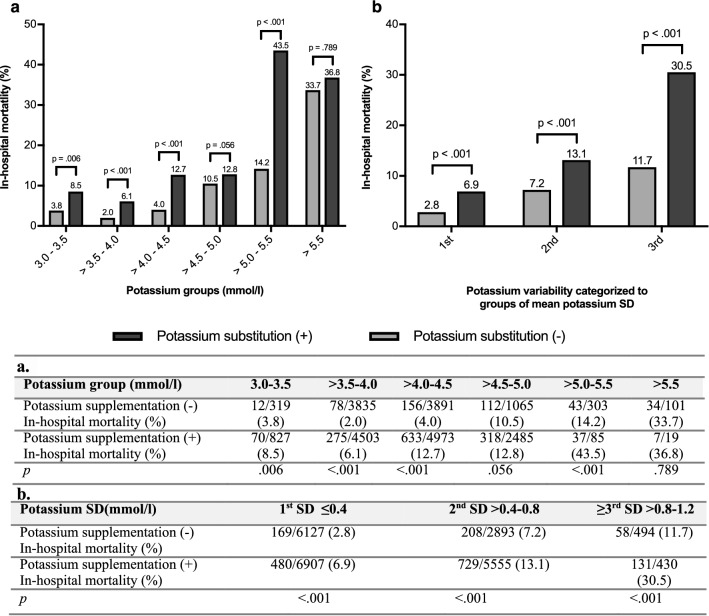
Association between potassium supplementation and in-hospital mortality, *n* = 22,406 patients. The table shows number of death and total number of patients in groups of (**a**) mean potassium concentration and (**b**) potassium variability. (+) Patients received potassium supplementation, (−) patients did not receive potassium supplementation. *p* values determined by *χ*^2^ -test

For this subanalysis, we included 22,406 patients, who were admitted to one of the ICUs between 2013 and 2018. 798 (5.2%) of these patients died during hospital stay. 12,892 (57.5%) patients received oral or intravenous potassium supplementation. Mean potassium concentration in patients receiving potassium supplementation (*p* = 0.001) and variability (*p* < 0.001) was significantly higher compared to patients without potassium supplementation (Additional file 1: Table S4). Potassium supplementation was associated with increased mortality (11.6%) compared to patients without potassium supplementation (4.8%, *p* < 0.001). This result is also present in the separate analysis of mean potassium concentrations between 3.0–3.5, > 3.5–4.0, > 4.0–4.5, and > 5.0–5.5 mmol/l (Fig. 4a). Lowest mortality rate (2.0%) was observed in patients with mean potassium concentrations between > 3.5 and 4.0 mmol/l who did not receive potassium supplementation. Potassium supplementation was not associated with a reduction of in-hospital mortality risk in any of the groups. In each group of potassium variability (1st, 2nd, ≥ 3rd SD), potassium supplementation was associated with a significant increase in in-hospital mortality (*p* < 0.001, Fig. 4b).

## Discussion

There is a lack of evidence for clear recommendations on potassium management in critically ill patients. We observed lowest mortality (3.7%) in ICU patients with a mean potassium range between > 3.5 and 4.0 mmol/l and low potassium variability. In-hospital mortality increased significantly for lower and higher potassium ranges including the normokalaemic range (3.5–5.0 mmol/l). More obvious, an increased potassium variability indicated by increased potassium concentration standard deviation or coefficient of variation during hospital stay is associated with an increased in-hospital mortality. In addition, we found an increased in-hospital mortality risk in ICU patients receiving potassium supplementation.

Our findings are in line with results of a recent systematic review, investigating the association of potassium concentration with mortality and occurrence of ventricular arrhythmias in patients after myocardial infarction in 12 studies. In those cardiac risk patients, they found an increased mortality risk when potassium concentrations were above 4.5 mmol/l [12]. In agreement, in patients with atrial fibrillation, we observed the lowest mortality when mean potassium was > 3.5–4.0 mmol/l. The few previous studies investigated the association between potassium ranges, variability and mortality in ICU patients reported a J- and U-shaped association between potassium and mortality [5, 13]. However, their classification lacked to define a tight potassium range with the lowest mortality. Our findings are in line with results reported by Hessels et al. and Uijtendaal et al. who showed that potassium variability was independently associated with outcome [5, 13]. McMahon et al. [6] investigated potassium concentrations at initiation of critical care in a comparable large ICU patient cohort. They stressed the importance of tight potassium regulation, as potassium concentrations between 4.5 and 5.5 mmol/l were associated with an increase in mortality risk. Unfortunately, they excluded patients with serum potassium concentrations < 4.0 mmol/l, which was in our analysis identified as the group with the lowest mortality. Unexpectedly, in our patient cohort, in-hospital mortality was significantly lower even in patients with mild hypokalaemic concentrations (3.0–3.5 mmol/l) as compared to 4.0–4.5 mmol/l. Contrary, in patients after myocardial infarction, potassium concentrations < 3.5 mmol/l were associated with an increased risk of ventricular arrhythmias [12].

Potassium supplementation in critically ill patients is usually performed intravenously with a high risk for causing severe hyperkalaemia [3, 14]. It may be an issue that potassium substitution is guided according to extracellular potassium measurements, which may not necessarily correlate with the intracellular potassium pool. In our cohort, mean potassium concentrations were slightly higher in patients receiving potassium supplementation. In addition, potassium substitution may increase potassium variability. We showed a significantly higher potassium variability in patients receiving potassium supplementation. Due to the lack of standardised protocols, decisions on when to start potassium supplementation are variable [15]. In our cohort, it was performed frequently, but was not associated with mortality reduction. Contrary, there was a tendency for an increased in-hospital mortality risk in patients who received potassium supplementation in each potassium group.

pH value is known as one strong confounding factor of potassium as metabolic acidosis causes a potassium shift from the intracellular to the extracellular space [16]. In our data, pH has a major impact on in-hospital mortality. After adjusting for age, sex and pH value in model 1 mean potassium levels and variability were independently associated with mortality. When evaluating multiple confounding factors in model 2, the results were not that clearly after adding pH value. Complex interactions in between the confounding factors may be responsible for a bias. Interestingly, when calculating the regression model divided by pH groups < 7.36, 7.36–7.44, > 7.44, low mean potassium levels (3.5–4.0 mmol/l) were confirmed as beneficial for the acidosis group (< 7.36). This indicates that especially in those patients, a mean potassium range > 3.5–4.0 mmol/l seems to be beneficial. This may be due to prevention of hyperkalaemia. A low potassium variability is associated with improved outcome after adjusting for multiple confounders including pH.

Interestingly, similar to our results, an association between mortality and mean glucose concentrations as well as glycaemic variability has been described by Krinsley [17–19]. In addition, hypoglycaemia is a risk factor for death in ICU patients [20]. However, the regression model 2 showed that our results are independent of mean, minimum or maximum glucose concentrations, as well as glycaemic variability, as risk factors for death [17–20]. Thus, the association between potassium categories and variability can be discussed in context of glucose control by insulin therapy, which is beneficial for critically ill patients with stress-induced hyperglycaemia [21–23]. In human physiology, the activation of Na^+^K^+^-ATPase by insulin is essential for the immediate potassium uptake to avoid hyperkalaemia for example after ingestion [24]. Insulin leads as a GLUT 4-independent effect to the activation of the Na^+^/K^+^-ATPase and reduction of potassium concentrations [25, 26]. Therefore, it may have underestimated positive effects on potassium concentrations as it may favour lower normokalaemic mean potassium concentrations, which were in our study associated with lower mortality. However, it remains unclear if glucose-independent insulin effects contribute to benefits of insulin therapy in ICU patients [27]. In critically ill rabbits, normoglycaemia in combination with elevated insulin concentrations improved myocardial contractility [27]. In the Leuven insulin trial, there were 6% more potassium measurements below 4.0 mmol/l, whilst hypokalaemia < 3.5 mmol/l was successfully avoided in the group targeting tight glucose concentrations 80–110 mg/dl [28]. Accordingly, the number of potassium measurements in the range of 3.5–4.0 mmol/l was slightly higher in the group with better survival.

Furthermore, insulin therapy may influence potassium variability. A recent trial, investigating potassium concentrations before and after implementing tight glycaemic control in the ICU, found a reduced potassium variability after implementation of tight glycaemic control [13]. Since glucose control by insulin goes along with changes in potassium concentrations, close monitoring of both, potassium and glucose, is of importance. A previous prospective study showed a reduction of hyper- and hypokalaemia in ICU patients using a computer-guided potassium regulation program [29]. For the future, a continuous monitoring system for both glucose and potassium in combination with an adequate protocol could be an interesting approach to target optimal glucose and potassium concentrations with a minimum variability.

## Limitations

This retrospective study shows associations but was not designed to prove causality. ICU patients in general were pooled together. However, there may be different potassium target ranges depending on the medical condition. Potassium concentrations have numerous interactions with medications, clinical and biological conditions, which were only partly considered in the multivariable regression model. The analysis of potassium supplementation has several limitations and potential biases. A major limitation is the use of mean potassium concentrations for group categorisation, whilst short-term hypokalaemic episodes are not reported. Thus, hypokalaemic episodes and not the potential subsequent potassium supplementation may have resulted in a mortality increase. Interestingly, initial potassium values are associated with in-hospital mortality in the same manner as mean potassium levels. in addition, detailed information on potassium doses as well as time point of potassium measurement and application are lacking. We performed an analysis on potassium supplementation but did not consider nutrition or other medications such as insulin, ACE-blockers, diuretics, β-antagonists or β-sympathomimetics.

## Conclusion

Clear potassium target ranges and recommendations for potassium supplementation have to be determined for ICU patients. Our findings indicate that ICU patients may benefit from a lower potassium target range between > 3.5 and 4.0 mmol/l and a minimal potassium variability. This may vary due to disease and pre-existing conditions. Potassium substitution should be applied with caution, only with a clear indication, under consideration of potassium variability, and stopped or at least reduced to maintain steady state in time when reaching the lower target. Future studies need to critically evaluated at which potassium range substitution should be initiated to avoid severe hypokalaemia and when to treat hyperkalaemia in ICU patients. Close trend monitoring of potassium levels may be useful to avoid elevated variability. Randomised controlled trials are needed to confirm our finding and may change clinical routine.

## Supplementary information

**Additional file 1: Table S1**. Baseline characteristics in categories of potassium variability. **Figure S1**. Mean potassium groups and in-hospital mortality using smaller cut points. **Figure S2**. Association between mean potassium levels and in-hospital mortality in patients with atrial fibrillation. **Figure S3**. Association of mean potassium levels and in-hospital mortality in patients receiving dialysis. **Figure S4**. First potassium value and in-hospital mortality. **Figure S5**. Potassium variability (SD) and in-hospital mortality using smaller cut points. **Figure S6**. Potassium variability shown in groups of coefficient of variation (CV). **Figure S7**. Combination of mean potassium concentrations and a variability determined in coefficient of variation. **Figure S8**. Adjusted odds ratios for in-hospital mortality (without pH value). **Table S2**. Regression in groups of pH value < 7.36, 7.36–7.44, > 7.44 (age, sex). **Table S3**. Regression in groups of pH value < 7.36, 7.36–7.44, > 7.44 (multiple confounders). **Table S4**. Mean potassium levels and variability in patients receiving potassium supplementation.

## Data Availability

Ethical restrictions prevent public sharing of data. Editors, reviewers and interested researchers should contact the corresponding author or dairesearchdata@charite.de to request data access.

## Abbreviations

ICU: Intensive Care Unit
SD: standard deviation
APACHE: acute physiology and chronic health evaluation II score
SOFA: sepsis-related organ failure assessment
AKI: acute kidney injury
CKD: chronic kidney disease
