# Supplementary material for: Association between potassium concentrations, variability and supplementation, and in-hospital mortality in ICU patients: a retrospective analysis
Source: Ann Intensive Care. 2019 Sep 5;9:100. doi: 10.1186/s13613-019-0573-0 (PMC6728107; doi:10.1186/s13613-019-0573-0)
Supplement: Supplementary file 1 — Additional file 1: Table S1. Baseline characteristics in categories of potassium variability. Figure S1. Mean potassium groups and in-hospital mortality using smaller cut points. Figure S2. Association between mean potassium levels and in-hospital mortality in patients with atrial fibrillation. Figure S3. Association of mean potassium levels and in-hospital mortality in patients receiving dialysis. Figure S4. First potassium value and in-hospital mortality. Figure S5. Potassium variability (SD) and in-hospital mortality using smaller cut points. Figure S6. Potassium variability shown in groups of coefficient of variation (CV). Figure S7. Combination of mean potassium concentrations and a variability determined in coefficient of variation. Figure S8. Adjusted odds ratios for in-hospital mortality (without pH value). Table S2. Regression in groups of pH value < 7.36, 7.36–7.44, > 7.44 (age, sex). Table S3. Regression in groups of pH value < 7.36, 7.36–7.44, > 7.44 (multiple confounders). Table S4. Mean potassium levels and variability in patients receiving potassium supplementation. [file 13613_2019_573_MOESM1_ESM.doc]

**Additional file 1**

**Association between potassium levels, variability and supplementation and in-hospital mortality in ICU patients: a retrospective analysis**

**Short title: Potassium and mortality in ICU patients**

Lilian Jo Engelhardt^1^, Felix Balzer^1^, Michael C. Müller^1^, Julius J. Grunow^1^, Claudia D. Spies^1^, Kenneth B. Christopher^2^, Steffen Weber-Carstens^1,3^, Tobias Wollersheim^1,3^

1. Department of Anesthesiology and Operative Intensive Care Medicine (CCM, CVK), Charité – Universitätsmedizin Berlin, corporate member of Freie Universität Berlin, Humboldt-Universität zu Berlin, and Berlin Institute of Health, Augustenburger Platz 1, D-13353 Berlin, Germany
2. Division of Renal Medicine, Brigham and Women's Hospital, Harvard Medical School, 75 Francis Street, Boston, Massachusetts, USA
3. Berlin Institute of Health (BIH), Anna-Louisa-Karsch-Str. 2, D-10178 Berlin, Germany

Correspondence: Dr. med. Tobias Wollersheim, Dept. of Anesthesiology and Operative Intensive Care Medicine, Campus Virchow Klinikum and Campus Mitte, Charité - Universitätsmedizin Berlin, Augustenburger Platz 1, 13353 Berlin, Germany.

E-mail: [tobias.wollersheim@charite.de](mailto:tobias.wollersheim@charite.de) fon: +49 (0)30-450 651808 fax: +49 (0)30-450 551019

The authors declare that they have no competing interests.

**Additional methods**

The definition of acute kidney injury due to ICD-codes has undergone a change in recent years. Current ICD-10-codes define acute kidney injury according to KDIGO *(Kidney Disease Improving Global Outcomes)-Guidelines.* ICD-10-diagnoses were determined by physicians depending on the guidelines valid at the time.

ICD-10-GM derived covariates are as followed:

Hypokalemia E87.6, Hyperkalemia E87.5, Chronic kidney disease N18.1-5, N18.8-9, I12.0, I13.1-2, N19, P96.0, R39.2, Z49.0-2, Z99.2, Acute kidney injury N17.0-2, N17.8-9, N99.0, N99.9, T79.5, Diabetes E10.0-9, E11.0-9, E12.0-9, E13.0-9, E14.0-9, Arterial hypertension I10.0-1, I10.9, I15.0-2, I15.8-9, Atrial flutter I48.0-4, I48.9.

**Additional results**

**Table S1** Baseline characteristics in categories of potassium variability, n=53248 patients.

| Characteristics | **1^st^ SD**  **≤0.4mmol/l** | **2^nd^ SD**  **>0.4-0.8mmol/l** | **≥3^rd^ SD**  **>0.8-1.2mmol/l** |
| --- | --- | --- | --- |
| Number of patients | 29848 | 21010 | 2390 |
| Age | 64 [51/74] | 63 [50/74] | 67 [56/75] |
| Gender (%) female/male | 12668 (42.4) /  17180 (57.6) | 9776 (46.5) /  11234 (53.5) | 1149 (48.1) /  1241 (51.9) |
| APACHE II | 11.00 [5.00/17.00] | 13.00 [6.00/19.00] | 14.00 [6.00/21.00] |
| SOFA Score (mean) | 1.50 [0.50/3.14] | 2.00 [0.67/4.00] | 2.00 [0.50/4.00] |
| SAPS II | 26.00 [14.00/37.00] | 30.00 [17.00/42.00] | 30.00 [16.00/44.00] |
| Mean glucose (mg/dl) | 122.00 [105.05/143.50] | 125.5 [106.72/149.67] | 124.00 [104.50/153.00] |
| Mean glucose SD (mg/dl) | 20.29 [11.49/31.20] | 25.54 [15.73/39.31] | 24.91 [13.75/41.55] |
| Glucose min (mg/dl) | 99.00 [87.00/118.00] | 95.00 [81.00/115.00] | 98.00 [81.00/124.00] |
| Glucose max (mg/dl) | 145.00 [116.00/187.00] | 158.00 [121.00/216.00] | 152.00 [119.00/205.00] |
| Sodium mean (mmol/l) | 139.00 [137.20/140.67] | 138.67 [136.67/140.54] | 138.50 [136.20/140.50] |
| Mean ph value | 7.40 [7.37/7.43] | 7.40 [7.36/7.43] | 7.38 [7.33/7.41] |
| Length of ICU stay (hours) | 33.80[17.20/95.60] | 46.30 [19.40/142.10] | 37.00 [16.85/104.65] |
| Patients with mechanical ventilation (%) | 7470/29750 (25.1) | 7324/20908 (35.0) | 760/2383 (31.9) |
| Patients receiving renal replacement therapy (%) | 1601/29750 (5.4) | 2776/20908 (13.2) | 486/2383 (20.4) |
| **ICD-10 Diagnoses number of patients with positive diagnosis/absolute numbers (in %)** | | | |
| Diabetes mellitus | 10260/29848 (34.4) | 8819/21010 (42.0) | 998/2390 (41.8) |
| CKD | 5334/29837 (17.9) | 5942/21005 (28.3) | 835/2390 (34.9) |
| AKI | 2705/29837 (9.1) | 4080/21005 (19.4) | 547/2390 (22.9) |
| Atrial fibrillation | 6375/29837 (21.4) | 5657/21005 (26.9) | 615/2390 (25.7) |
| Hypertension | 15873/29837 (53.2) | 12337/21005 (58.7) | 1355/2390 (56.7) |
| Hypokalemia | 10849/29837 (36.4) | 10311/21005 (49.1) | 1042/2390 (43.6) |
| Hyperkalemia | 662/29837 (2.2) | 1528/21005 (7.3) | 437/2390 (18.3) |

**Table S1** Results are expressed as median with interquartile range or as absolute numbers with percentages. Abbreviations: Sequential organ failure assessment (SOFA), Acute physiology and chronic health evaluation (APACHE) II Score at ICU admission, Simplified Acute Physiology Score (SAPS), Intensive care unit (ICU), Acute kidney injury (AKI), Chronic kidney disease (CKD). Missing values are <2% and considered as not relevant or indicated. Glucose values are available in n=43694 patients; glucose variability was determined in n=33031 patients. pH values are available in n=30558 patients. Kruskal-Wallis-Test p<.001 in each category.

| **Mean potassium group (mmol/l)** | **Number of death/ number of patients** | **Mortality rate (%)** |
| --- | --- | --- |
| 3.0 | 2/29 | 6.9 |
| 3.1 | 23/215 | 10.7 |
| 3.2 | 28/340 | 8.2 |
| 3.3 | 43/641 | 6.7 |
| 3.4 | 80/1037 | 7.7 |
| 3.5 | 87/1771 | 4.9 |
| 3.6 | 129/2586 | 5.0 |
| 3.7 | 174/3673 | 4.7 |
| 3.8 | 200/4682 | 4.3 |
| 3.9 | 267/5504 | 4.9 |
| 4.0 | 283/5592 | 5.1 |
| 4.1 | 339/5104 | 6.6 |
| 4.2 | 340/4569 | 7.4 |
| 4.3 | 340/3721 | 9.1 |
| 4.4 | 294/3434 | 8.6 |
| 4.5 | 251/3043 | 8.2 |
| 4.6 | 234/2496 | 9.4 |
| 4.7 | 189/1653 | 11.4 |
| 4.8 | 149/1018 | 14.6 |
| 4.9 | 83/624 | 13.3 |
| 5.0 | 62/444 | 14.0 |
| 5.1 | 54/283 | 19.1 |
| 5.2 | 42/231 | 18.2 |
| 5.3 | 25/138 | 18.1 |
| 5.4 | 14/97 | 14.4 |
| 5.5 | 18/87 | 20.7 |
| 5.6 | 17/69 | 24.6 |
| 5.7 | 6/39 | 15.4 |
| 5.8 | 15/37 | 40.5 |
| 5.9 | 6/21 | 28.6 |
| 6.0 | 7/19 | 36.8 |

**Figure S1 Mean potassium groups and in-hospital mortality using smaller cut points,** n=53248 patients. Mean potassium levels between 3.5 and 4.0mmol/l are associated with the lowest mortality.

| Potassium group (mmol/l) | **3.0-3.5** | **>3.5-4.0** | **>4.0-4.5** | **>4.5-5.0** | **>5.0-5.5** | **>5.5** |
| --- | --- | --- | --- | --- | --- | --- |
| Number of patients | 497 | 3557 | 5521 | 2751 | 262 | 59 |
| Number of death | 62 | 297 | 632 | 397 | 71 | 21 |
| Mortality rate (%) | 12.5 | 8.3 | 11.4 | 14.4 | 27.1 | 35.6 |

**Figure S2 Association between mean potassium levels and in-hospital mortality in patients with atrial fibrillation.** In 12467 patients with atrial fibrillation mean potassium levels were 4.2 mmol/l [3.9/4.5], mean potassium SD was 0.40 mmol/l [0.29/0.52]. 1480 (11.7%) died during hospital stay. Lowest mortality was observed in patients with mean potassium levels between >3.5 and 4.0 mmol/l.

| Potassium group(mmol/l) | **3.0-3.5** | **>3.5-4.0** | **>4.0-4.5** | **>4.5-5.0** | **>5.0-5.5** | **>5.5** |
| --- | --- | --- | --- | --- | --- | --- |
| Dialysis (+) | 41/93  (44.1) | 204/798  (25.6) | 603/1995  (30.2) | 414/1457  (28.4) | 101/394  (25.6) | 35/126  (27.8) |
| Dialysis (-) | 197/3226 (6.1) | 756/19923(3.8) | 975/18520(5.3) | 380/5818(6.5) | 61/554(11.0) | 43/137(31.4) |

**Figure S3 Association of mean potassium levels and in-hospital mortality in patients receiving dialysis**. Patients with receiving dialysis (n = 4863) compared to patients without renal replacement therapy

(n=48187)

| **Kalium first (in mmol/l)** | **Mortality rate (%)** | **n**  **total** | **n death** |
| --- | --- | --- | --- |
| <3.0 | 12.8 | 1080 | 138 |
| 3.0-3.5 | 8.9 | 5475 | 489 |
| 3.5-4.0 | 5.6 | 18411 | 1034 |
| 4.0-4.5 | 6.0 | 17743 | 1069 |
| 4.5-5.0 | 9.2 | 6595 | 604 |
| 5.0-5.5 | 11.6 | 2242 | 260 |
| >5.5 | 13.5 | 1702 | 229 |

**Figure S4 First potassium value and in-hospital mortality, n=53248 patients**. Lowest mortality between >3.5-4.0mmol/l (5.6%).

| **Potassium SD group (mmol/l)** | **Number of death/ Number of patients** | **Mortality rate (%)** |
| --- | --- | --- |
| 0 | 62/831 | 7.5 |
| 0.1 | 198/4240 | 4.7 |
| 0.2 | 270/6921 | 3.9 |
| 0.3 | 542/11509 | 4.7 |
| 0.4 | 841/11750 | 7.2 |
| 0.5 | 664/7699 | 8.6 |
| 0.6 | 488/4688 | 10.4 |
| 0.7 | 259/2407 | 10.8 |
| 0.8 | 182/1356 | 13.4 |
| 0.9 | 112/675 | 16.6 |
| 1.0 | 68/387 | 17.6 |
| 1.1 | 37/264 | 14.0 |
| 1.2 | 27/146 | 18.5 |

**Figure S5 Potassium variability (SD) and in-hospital mortality using smaller cut points**. n= 53248 patients. Potassium SDs between 0.1 and 0.3mmol/l are associated with the lowest in-hospital mortality

|  | **CV 1**  **(0-10%)** | **CV 2**  **(10-20%)** | **CV 3**  **(20-30%)** | **CV 4**  **>30%** |
| --- | --- | --- | --- | --- |
| Mortality rate (%) | 5.4 | 9.1 | 13.9 | 21.0 |
| Total Numbers | 1697/31186 | 1814/19966 | 251/1806 | 61/290 |

**Figure S6 Potassium variability shown in groups of coefficient of variation (CV), n=53248.**

|  | **3.0-3.5** | **>3.5-4.0** | **>4.0-4.5** | **>4.5-5.0** | **>5.0-5.5** | **>5,5** |
| --- | --- | --- | --- | --- | --- | --- |
|  | Mortality rate in (%) | | | | | |
| CV 1 (0-10%) | 5.8 | 3.6 | 5.9 | 8.2 | 19.3 | 41.3 |
| CV2 (10-20%) | 7.1 | 5.8 | 10.2 | 13.8 | 15.8 | 26.6 |
| CV 3 (20-30%) | 12.6 | 12.6 | 14.3 | 15.1 | 17.9 | 18.9 |
| CV 4 (>30%) | 24.2 | 18.8 | 23.7 | 20.0 | 14.3 | 26.3 |
|  | Total numbers | | | | | |
| CV 1 (0-10%) | 81/1385 | 460/12888 | 734/12535 | 325/3966 | 64/332 | 33/80 |
| CV2 (10-20%) | 118/1661 | 420/7258 | 760/7479 | 411/2978 | 76/481 | 29/109 |
| CV 3 (20-30%) | 32/254 | 75/596 | 75/526 | 45/298 | 17/95 | 7/37 |
| CV 4 (>30%) | 8/33 | 9/48 | 14/59 | 14/70 | 6/42 | 10/38 |

**Figure S7 Combination of mean potassium concentrations and a variability determined in coefficient of variation, n=53248 patients.** Potassium concentrations between 3.5 and 4.0 mmol/l and a variability in CV group 1 were associated with the lowest mortality (3.6%).

|  | **Unadjusted** | | | **Model 1** | | |  | **Model 2** | | | | **20-fold imputation**  **for missing values** | | |
| --- | --- | --- | --- | --- | --- | --- | --- | --- | --- | --- | --- | --- | --- | --- |
|  | **OR** | **95% CI** | ***p*** | **OR** | **95% CI** | ***p*** |  | **OR** | | **95% CI** | ***p*** | **OR** | **95% CI** | ***p*** |
| **Potassium groups (in mmol/l)** | | | | | | | | | | | | | | |
| 3.0-3.5 | 1.49 | 1.29-1.73 | <.001 | 1.49 | 1.29-1.73 | <.001 | 3.0-3.5 | 1.54 | | 1.24-1.91 | <.001 | 1.58 | 1.34-1.88 | <.001 |
| >3.5-4.0 | 1.00 |  |  | 1.00 |  |  | >3.5-4.0 | 1.00 | |  |  | 1.00 |  |  |
| >4.0-4.5 | 1.60 | 1.48-1.74 | <.001 | 1.51 | 1.39-1.64 | <.001 | >4.0-4.5 | 1.18 | | 1.05-1.32 | 0.005 | 1.14 | 1.03-1.26 | 0.009 |
| >4.5-5.0 | 2.09 | 1.89-2.30 | <.001 | 1.90 | 1.71-2.10 | <.001 | >4.5-5.0 | 1.19 | | 1.04-1.37 | 0.014 | 1.21 | 1.07-1.37 | 0.003 |
| >5.0-5.5 | 2.94 | 2.44-3.55 | <.001 | 2.86 | 2.37-3.45 | <.001 | >5.0-5.5 | 2.44 | | 1.89-3.16 | <.001 | 2.38 | 1.89-3.01 | <.001 |
| >5.5 | 5.40 | 4.07-7.16 | <.001 | 5.67 | 4.26-7.55 | <.001 | >5.5 | 6.73 | | 4.54-9.98 | <.001 | 6.88 | 4.88-9.71 | <.001 |
| **Potassium variability** | | | | | | | | | | | | | | |
| 1^st^ SD | 1.00 |  |  | 1.00 |  |  | 1^st^ SD | 1.00 | |  |  | 1.00 |  |  |
| 2^nd^ SD | 1.75 | 1.63-1.88 | <.001 | 1.73 | 1.61-1.86 | <.001 | 2^nd^ SD | 1.26 | | 1.15-1.38 | <.001 | 1.20 | 1.10-1.31 | <.001 |
| ≥3^rd^ SD | 2.74 | 2.41-3.11 | <.001 | 2.79 | 2.45-3.17 | <.001 | ≥3^rd^ SD | 1.88 | | 1.57-2.24 | <.001 | 1.84 | 1.57-2.15 | <.001 |
| |  | | --- | |  |  |  |  |  |  | **Covariates** | | | | | | | |
|  |  |  |  |  |  |  | Age | | 1.02 | 1.01-1.02 | <.001 | 1.02 | 1.01-1.02 | <.001 |
|  |  |  |  |  |  |  | Gender | | 0.97 | 0.89-1.06 | 0.497 | 0.98 | 0.92-1.04 | 0.579 |
|  |  |  |  |  |  |  | Glucose mean | | 1.00 | 1.00-1.01 | 0.051 | 1.00 | 1.00-1.00 | 0.158 |
|  |  |  |  |  |  |  | Glucose SD | | 1.00 | 1.00-1.00 | 0.573 | 1.00 | 1.00-1.00 | 0.950 |
|  |  |  |  |  |  |  | Glucose minimum | | 1.00 | 1.00-1.00 | 0.079 | 1.00 | 1.00-1.00 | 0.092 |
|  |  |  |  |  |  |  | Glucose maximum | | 1.00 | 1.00-1.00 | 0.151 | 1.00 | 1.00-1.00 | 0.202 |
|  |  |  |  |  |  |  | Sodium mean | | 1.04 | 1.03-1.05 | <.001 | 1.04 | 1.03-1.05 | <.001 |
|  |  |  |  |  |  |  | Sodium SD | | 1.18 | 1.15-1.21 | <.001 | 1.18 | 1.16-1.21 | <.001 |
|  |  |  |  |  |  |  | APACHE II (ICU admission) | | 1.04 | 1.03-1.04 | <.001 | 1.04 | 1.04-1.04 | <.001 |
|  |  |  |  |  |  |  | SOFA Score maximum | | 1.00 | 0.99-1.01 | 0.438 | 1.00 | 0.99-1.01 | 0.433 |
|  |  |  |  |  |  |  | Diabetes | | 1.03 | 0.93-1.13 | 0.578 | 1.11 | 1.02-1.20 | 0.021 |
|  |  |  |  |  |  |  | AKI | | 4.87 | 4.42-5.37 | <.001 | 5.01 | 4.60-5.44 | <.001 |
|  |  |  |  |  |  |  | CKD | | 0.70 | 0.63-0.77 | <.001 | 0.73 | 0.66-0.80 | <.001 |
|  |  |  |  |  |  |  | Atrial fibrillation | | 1.10 | 0.99-1.21 | 0.070 | 1.08 | 0.99-1.18 | .082 |
|  |  |  |  |  |  |  | ICU length of stay (hours) | | 1.00 | 1.00-1.00 | <.001 | 1.00 | 1.00-1.00 | <.001 |
|  |  |  |  |  |  |  | Number of measurements | | 1.00 | 1.00-1.00 | 0.006 | 1.00 | 1.00-1.00 | <.001 |

**Figure S8 Adjusted odds ratios for in-hospital mortality (without pH value).** Reference for potassium categories is >3.5-4.0mmol/l, for potassium variability 1st SD. Unadjusted: n= 53248 patients. Adjusted model 1: n= 53248 patients, adjusted for gender and age. Adjusted model 2: n=32594 patients, 20654 missing values, adjusted for gender, age, glucose mean, glucose SD, glucose maximum, glucose minimum, sodium mean, sodium SD, APACHE II Score, SOFA Score maximum, diabetes, acute kidney injury (AKI), chronic kidney disease (CKD), atrial fibrillation. Corrected for missing values by twenty-fold imputations. Blood glucose concentrations in mg/dl and sodium concentration in mmol/l. Forest-Plot of original data from model 2.

**Table S2 Regression in groups of pH value <7.36, 7.36-7.44, >7.44 (age, sex)**

|  | **pH < 7.36, n=6828** | | | **pH 7.36-7.44, n=19234** | | | **pH > 7.44, n= 4496** | | |
| --- | --- | --- | --- | --- | --- | --- | --- | --- | --- |
| **Potassium in mmol/l** | OR | 95% CI | p | OR | 95% CI | p | OR | 95% CI | p |
| **3.0-3.5** | 1.52 | 0.97-2.38 | 0.071 | 1,70 | 1.24-2.34 | 0.001 | 1.66 | 1.15-2.40 | 0.007 |
| **>3.5-4.0** |  |  | <.001 |  |  | <.001 |  |  | 0.005 |
| **>4.0-4.5** | 1.67 | 1.35-2.06 | <.001 | 1.53 | 1.31-1.77 | <.001 | 1.39 | 1.10-1.77 | 0.007 |
| **>4.5-5.0** | 2.28 | 1.81-2.86 | <.001 | 1.45 | 1.21-1.73 | <.001 | 1.84 | 1.30-2.62 | 0.001 |
| **>5.0-5.5** | 2.85 | 2.08-3.91 | 0.001 | 1.98 | 1.31-2.99 | 0.001 | 1.83 | 0.68-4.92 | 0.234 |
| **>5.5** | 7.11 | 4.65-10.86 | <.001 | 3.09 | 1.45-6.60 | 0.004 | 0.00 | 0.00 | 0.999 |
| **1^st^ SD** |  |  | <.001 |  |  | <.001 |  |  | <.001 |
| **2^nd^ SD** | 1.85 | 1.58-2.18 | <.001 | 1.99 | 1.76-2.25 | <.001 | 1.29 | 1.04-1.61 | 0.02 |
| **≥3^rd^ SD** | 2.63 | 2.05-3.37 | <.001 | 2.60 | 1.99-3.40 | <.001 | 2.26 | 1.48-3.46 | <.001 |
| **Age** | 1.03 | 1.02-1.04 | <.001 | 1.02 | 1.02-1.03 | <.001 | 1.01 | 1.01-1.02 | 0.001 |
| **Gender** | 1.08 | 0.93-1.25 | 0.338 | 1.00 | 0.89-1.13 | 0.968 | 1.24 | 1.00-1.54 | 0.049 |

**Table S3 Regression in groups of pH value <7.36, 7.36-7.44, >7.44 (multiple confounders)**

|  | pH < 7.36, n=5485 | | | pH 7.36-7.44, n= 16498 | | | pH > 7.44, n= 3653 | | |
| --- | --- | --- | --- | --- | --- | --- | --- | --- | --- |
| Potassium in mmol/l | OR | 95% CI | p | OR | 95% CI | p | OR | 95% CI | p |
| 3.0-3.5 | 2.21 | 1.31-3.72 | 0.003 | 1.45 | 0.98-2.16 | 0.066 | 1.39 | 0.86-2.24 | 0.180 |
| >3.5-4.0 | 1.00 |  |  | 1.00 |  |  | 1.00 |  |  |
| >4.0-4.5 | 1.41 | 1.09-1.82 | 0.009 | 1.11 | 0.92-1.32 | 0.275 | 1.07 | 0.80-1.43 | 0.638 |
| >4.5-5.0 | 1.77 | 1.33-2.37 | <.001 | 0.90 | 0.73-1.12 | 0.357 | 1.33 | 0.85-2.06 | 0.209 |
| >5.0-5.5 | 2.49 | 1.66-3.75 | <.001 | 2.34 | 1.42-3.87 | .001 | 2.71 | 0.77-9.52 | 0.120 |
| >5.5 | 8.84 | 5.14-15.22 | <.001 | 4.26 | 1.70-10.65 | .002 | 0.00 | 0.00-0.00 | 0.999 |
| 1^st^ SD | 1.00 |  |  | 1.00 |  |  | 1.00 |  |  |
| 2^nd^ SD | 1.25 | 1.02-1.53 | 0.035 | 1.28 | 1.11-1.48 | 0.001 | 1.04 | 0.80.-1.35 | 0.769 |
| ≥3^rd^ SD | 1.53 | 1.12-2.10 | 0.008 | 1.75 | 1.26-2.42 | 0.001 | 2.04 | 1.19-3.48 | 0.009 |
| Age | 1.03 | 1.02-1.03 | <.001 | 1.02 | 1.01-1.02 | <.001 | 1.01 | 1.00-1.02 | 0.017 |
| Gender | 1.12 | 0.93-1.34 | 0.229 | 0.92 | 0.80-1.06 | 0.255 | 1.05 | 0.82-1.35 | 0.712 |
| Glucose mean | 1.00 | 1.00-1.01 | 0.276 | 1.00 | 1.00-1.01 | 0.421 | 1.01 | 1.00-1.02 | 0.038 |
| Glucose SD | 0.99 | 0.98-0.99 | <.001 | 1.00 | 0.99-1.01 | 0.702 | 1.00 | 0.99-1.01 | 0.893 |
| Glucose minimum | 0.99 | 0.99-1.00 | <.001 | 1.00 | 1.00-1.00 | 0.751 | 1.00 | 0.99-1.00 | 0.372 |
| Glucose maximum | 1.01 | 1.00-1.01 | <.001 | 1.00 | 1.00-1.00 | 0.598 | 1.00 | 0.99-1.00 | 0.017 |
| Sodium mean | 1.04 | 1.01-1.06 | 0.004 | 1.06 | 1.04-1.08 | <.001 | 1.03 | 1.00-1.06 | 0.049 |
| Sodium SD | 1.11 | 1.06-1.17 | <.001 | 1.25 | 1.20-1.30 | <.001 | 1.12 | 1.05-1.9 | 0.001 |
| APACHE II (ICU admission) | 1.04 | 1.04-1.05 | <.001 | 1.04 | 1.03-1.04 | <.001 | 1.04 | 1.02-1.05 | 0.000 |
| SOFA Score maximum | 0.99 | 0.97-1.01 | 0.505 | 1.00 | 0.99-1.02 | 0.841 | 0.98 | 0.96.1.01 | 0.248 |
| Diabetes | 0.84 | 0.69-1.03 | 0.093 | 1.11 | 0.90-1.23 | 0.530 | 1.50 | 1.14-1.98 | 0.004 |
| AKI | 5.35 | 4.4-6.5 | <.001 | 4.47 | 3.85-5.19 | <.001 | 3.38 | 2.56-4.46 | 0.000 |
| CKI | 0.43 | 0.36-0.53 | <.001 | 0.770 | 0.66-0.90 | 0.001 | 0.56 | 0.41-0.75 | 0.000 |
| Atrial fibrillation | 1.31 | 1.07-1.61 | .008 | 1.080 | 0.93- 1.25 | 0.313 | 0.96 | 0.74-1.26 | 0.776 |
| ICU LOS (in h) | 1.00 | 1.00-1.00 | 0.925 | 1.000 | 1.00-1.00 | <.001 | 1.00 | 1.00-1.00 | 0.094 |
| N potassium measurements | 1.00 | 1.00-1.00 | 0.142 | 1.000 | 1.00-1.00 | 0.906 | 1.00 | 1.00-1.00 | 0.578 |

**Subanalysis potassium supplementation 2013-2018**

**Table S4 Mean potassium levels and variability in patients receiving potassium supplementation**

|  | Mean potassium level | Mean potassium SD | Number of patients |
| --- | --- | --- | --- |
| No potassium supplementation (-) | 4.06 [3.85/4.33] | 0.33[0.22/0.47] | 9514 |
| Potassium supplementation (+) | 4.10 [3.83/4.44] | 0.39 [0.30/0.49] | 12892 |
| *p (Mann-Whitney-U-test)* | .001 | <.001 |  |
